# Effects of short-duration spaceflight on the execution of critical mission tasks

**DOI:** 10.3389/fphys.2025.1677377

**Published:** 2025-09-29

**Authors:** Gilles Clément, Sarah Moudy, Timothy R. Macaulay, Scott J. Wood

**Affiliations:** ^1^ KBR, Houston, TX, United States; ^2^ Aegis Aerospace, Houston, TX, United States; ^3^ Neuroscience Laboratories, NASA Johnson Space Center, Houston, TX, United States

**Keywords:** functional performance, spaceflight, sensorimotor system, vestibular tests, private astronaut mission

## Abstract

**Background:**

The objective of this study was to investigate how short-duration spaceflight affects private astronauts’ performance of mission-critical functional tasks that challenge balance and locomotor control systems shortly after they return to Earth.

**Methods:**

Ten astronauts were assessed while they performed three functional tests (sit-to-stand, tandem walk, and walk-and-turn) before spaceflight and a few hours after returning from missions lasting from 4 to 21 days. Their performance was compared to that of 36 astronauts who returned from long-duration missions lasting from 6 to 12 months.

**Results:**

Shortly after return from a short-duration spaceflight, astronauts had difficulty standing, walking, and turning around obstacles, and they experienced terrestrial readaptation motion sickness. However, the performance of these functional tasks was less impacted after short-duration missions than after long-duration missions. After long-duration spaceflight, astronauts took longer to stabilize when standing, made fewer correct steps in balance tests (especially with eyes closed), needed more time for walking tasks, and turned more slowly than after short-duration flight. Motion sickness ratings were more variable and often higher in the long-duration group.

**Conclusion:**

Similar to long-duration spaceflight, short-duration missions can also result in significant postflight vestibular and sensorimotor impairments, potentially affecting the ability of some crewmembers to perform critical mission tasks.

## Introduction

Astronauts returning from long-duration stays on the International Space Station (ISS) often experience disorientation, perceptual illusions, and re-entry motion sickness. Upon re-exposure to Earth’s gravity, they face functional challenges such as postural imbalances (Wood et al., 2015) and reduced dynamic control of postural stability (Miller et al., 2018; Mulavara et al., 2018). These effects are most pronounced during gravity transitions, which coincide with critical operational tasks like landing and emergency egress (Clément et al., 2022). As a result, testing in this period should focus on tasks that reflect high-priority exploration mission demands (Paloski et al., 2008).

To evaluate the effects of vestibular and sensorimotor adaptation, NASA is currently collecting a set of targeted measures on astronauts after they return from spaceflights of varying durations. These measurements, referred to as sensorimotor standard measures, are designed to be efficient, requiring minimal time and logistical resources. The selection of 3 specific functional tests—sit-to-stand, tandem walk, and walk-and-turn—was guided by the need to evaluate performance on tasks that are critical operationally and that challenge sensorimotor control after return to Earth.

Astronauts must be able to effectively perform functional tasks after long-duration spaceflight to support operations such as lunar or Martian landings, surface activities, and vehicle egress upon return to Earth. After short-duration commercial spaceflights, private astronauts must also be able to perform functional tasks that allow them to safely egress the vehicle, including during potential emergencies. A subset of the data from 25 astronauts performing these 3 functional tasks after multi-month missions has been previously reported (Clément et al., 2022; Clément et al., 2023). The aim of the present study was to assess how short-duration astronauts perform these same tasks a few hours after returning from spaceflights lasting only a few days to a few weeks.

## Methods

### Participants

Two groups of participants took part in this study. The first group, referred to as the *short-duration* group, consisted of 10 healthy individuals (6 males, 4 females; mean age = 45.9 years, standard deviation [SD] = 13.3 years) who completed an Axiom, Fram2, or Soyuz taxi mission lasting from 4 to 21 days (mean = 10.4 days, SD = 7.4 days). Eight of these individuals were first-time flyers, whereas the other 2 individuals had previously completed multiple 6-month missions in space. The second group, referred to as the *long-duration* group, included 36 healthy individuals (22 males, 14 females; mean age = 47.7 years, SD = 8.5 years) who completed an ISS mission lasting from 5 to 12 months (mean = 6.8 months, SD = 1.7 months) on board the ISS. The long-duration group included 19 first time flyers and 17 repeat flyers. In a previous publication (Clément et al., 2022), we found no significant difference between first-time flyers and the experienced flyers for the measures collected during these tasks.

All participants passed a flight physical medical examination and had no known history of vestibular or oculomotor abnormalities. The test procedures were approved by the NASA Institutional Review Board and were performed in accordance with the ethical standards outlined in the 1964 Declaration of Helsinki. All participants provided a written informed consent before participating in the study. Participants provided consent to publish identifying information and images in an online open-access publication.

Participants completed 3 functional tests: sit-to-stand, tandem walk, and walk-and-turn. A familiarization session and a data collection session were conducted on average 67 ± 19 days before launch for the short-duration group and 98 ± 88 days before launch for the long-duration group. Trained operators tested the short-duration group on average 3.6 ± 1.1 h after landing (median: 3.8 days, Q1: 3.4 days, Q3: 4.1 days), and the long-duration group 4.5 ± 5.2 h after landing (median: 3.0 days, Q1: 2.5 days, Q3: 3.4 days), depending on location and recovery operations. During each test, participants wore a triaxial inertial measurement unit (IMU) (Opal V2 or Emerald, APDM Inc., Portland, OR, United States) secured to their trunk with an elastic strap. GoPro cameras were used to continuously record the scene and the participants as they performed the functional tests.

## Sit-to-stand

**FIGURE 1:**
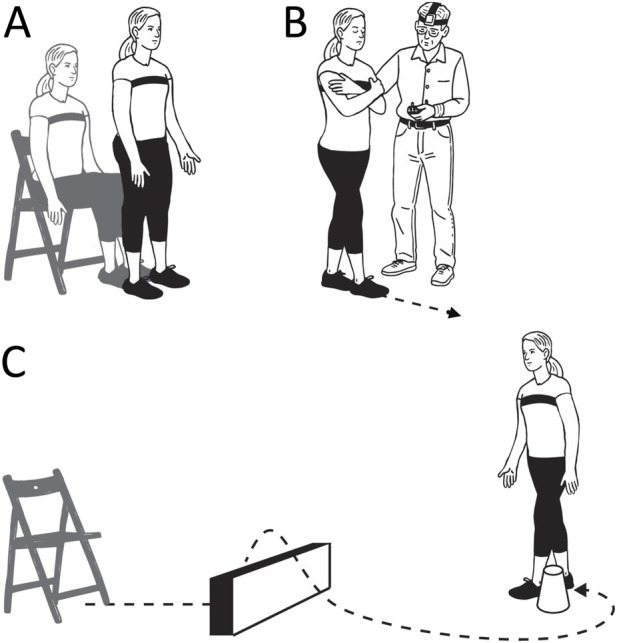
Functional tests. Sit-to-stand **(A)**, tandem walk **(B)**, and walk-and-turn **(C)**. Credit: Olga Kuldavletova.

Participants were instructed to stand up from a seated position as quickly as possible without using their hands and to maintain a quiet standing posture for 10 s (Figure 1A). Each subject completed 2 trials. Performance was quantified by measuring the time elapsed between the command to stand and the attainment of a stable posture. This transition was identified using data from the IMU.

The onset and completion of the standing movement were determined using the absolute angular velocity of the participants’ trunks in the pitch plane. The sitting phase served as the baseline and the first point at which the signal exceeded 5 times the standard deviation of this baseline was used to establish the initiation of movement (Clément et al., 2022). A second baseline was calculated from the signal during the final 2 s of the 10-s standing period. The end of the stand was established as the first time point at which the signal dropped below 5 times the standard deviation of this second baseline and remained below that threshold for at least one second. All data were visually inspected and manually verified for accuracy.

## Walk-and-turn

The walk-and-turn test was conducted after the sit-to-stand test. Starting from a standing position in front of the chair, participants were instructed to walk in a straight line toward a cone placed 4 m away, walk around it, return to the starting point, and sit down in the chair. The participants had to step over a 30-cm-high obstacle during both their outbound and return paths (Figure 1C). The entire sequence was repeated twice. The walk-and-turn test was added later in the study, so it was completed by only 25 participants from the long-duration group and 9 from the short-duration group.

Performance was evaluated based on the total time to complete the task and the yaw angular velocity of the participants’ trunks as they navigated around the 180-deg cone turn. Throughout the entire task, the resultant acceleration was computed as the root sum of squares of the acceleration signals along the x-, y-, and z-axes. The beginning and end of the walking phase were identified when acceleration exceeded or fell below, respectively, 5 times the standard deviation of the baseline measurement.

The turning rate was calculated exclusively during the cone navigation. The mean and standard deviation of the yaw angular velocity of the trunk were established during the straight walking segments. The start of the cone turn was detected when the yaw velocity surpassed 5 times this standard deviation threshold. The turn was considered complete once the participant had rotated more than 165° relative to their orientation at the turn’s onset (Clément et al., 2023).

### Tandem walk

The participants completed the tandem walk test either before the sit-to-stand test or after the walk-and-turn test. The tandem-walk test is commonly employed in clinical settings to evaluate dynamic balance control in individuals who are at risk of falling (Cohen et al., 2012). Participants were instructed to take 10 heel-to-toe steps with their arms crossed over their chest, completing 2 trials with their eyes closed and 2 trials with their eyes open (Figure 1B). Each trial was video recorded, and foot position data were captured using a pressure sensing walkway (Zeno Walkway, ZenoMetrics, Peekskill, NY, United States). Because the pressure-sensing walkway was not available at all data collection sessions, two analysis methods were used. When the walkway was unavailable, three trained reviewers independently scored the percentage of correct steps based on video recordings of the crewmembers’ foot movements. A “misstep” was defined by any of the following criteria: (a) the stepping foot crossing over the stationary foot; (b) stepping sideways before completing a step; (c) the stepping foot moving in a wide, arcing trajectory before placement; or (d) a gap larger than 10 cm between the heel of the front foot and the toe of the back foot at step completion (Miller et al., 2018; Mulavara et al., 2018).

When walkway data were available, an automated detection algorithm was used to calculate the percentage of steps meeting the predefined criteria. A single independent reviewer verified the algorithm outputs, and additional reviewers were consulted when necessary. For trials evaluated with both analysis methods, the difference in scores was 6%, demonstrating a high level of consistency between the two approaches.

Once all assessments were complete, either the algorithm’s score or the median of three reviewers’ scores was used to determine the percentage of correct steps per trial, with higher percentages reflecting better performance.

### Terrestrial readaptation motion sickness

Before and after each of the 3 functional tests, crewmembers were asked to rate their level of motion sickness on a scale from 0 to 20, where 20 indicates vomiting and 10 represents a level halfway to vomiting.

### Statistical analysis

The 6 outcome measures included: (a) the average time required to complete both trials of the sit-to-stand test; (b) the average percentage of correct steps across both trials of the tandem walk with eyes open; (c) the average percentage of correct steps across both trials of the tandem walk with eyes closed; (d) the average time to complete both trials of the walk-and-turn test; (e) the average turn rate during both trials of the walk-and-turn test; and (f) the average re-adaptation motion sickness scores before and after performing the 3 functional tests.

Terrestrial readaptation motion sickness, orthostatic intolerance, or mission-related constraints prevented 4 of the individuals in the long-duration group (11.1%) from completing some or all of the functional tests shortly after landing. If a participant was physically unable to complete the test after flight, the worst score recorded among other participants was assigned to them to better represent the data. This imputation was applied to 6 individuals in the long-duration group and none in the short-duration group.

A two-way linear mixed model was used for comparing changes due to spaceflight timepoint (Pre vs. Post) and differences in the outcome measures between the short-duration and long-duration groups. Another linear mixed model was used for examining sex differences across outcome measures. Both analyses were conducted using MATLAB R2023b MathWorks, Natick, MA). In the Results section, data are expressed as median values with interquartile ranges (Q1–Q3).

## Results

**FIGURE 2:**
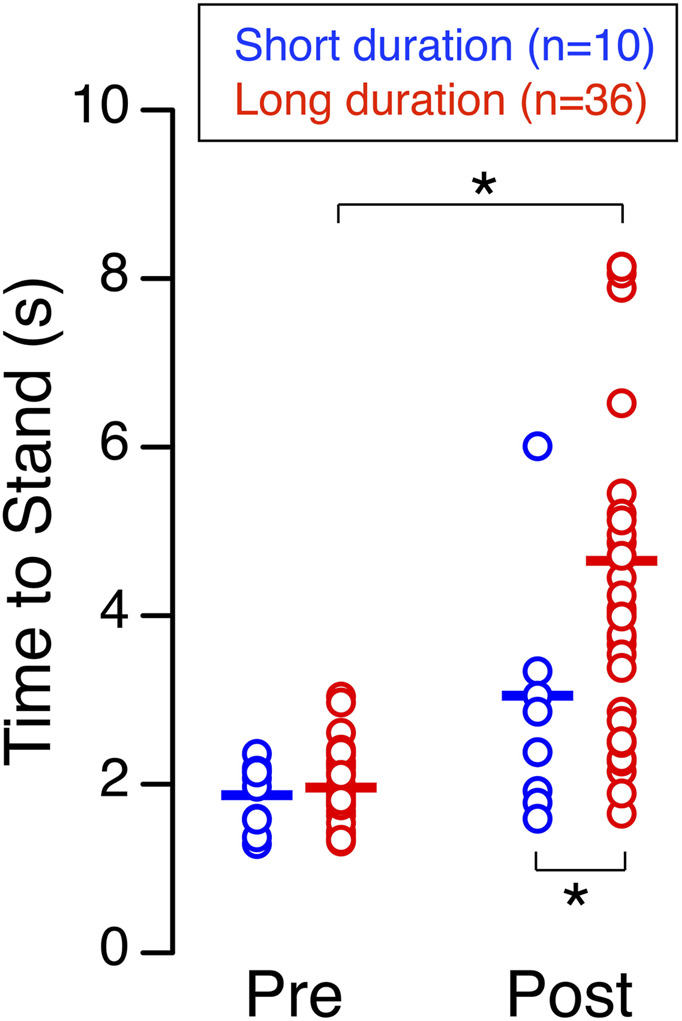
Sit-to-stand. Time required to achieve a stable standing posture from a seated position before (Pre) and shortly after (Post) spaceflight for 10 astronauts who participated in short-duration missions and 36 astronauts who participated in long-duration missions. Each symbol represents an individual subject, and the horizontal line indicates the group mean. *p < 0.05.

Participants in the long-duration group required a significantly longer time to achieve a stable posture during the sit-to-stand test shortly after landing (4.24 s, 2.86–5.45 s), than they did before flight (1.89 s, 1.75–2.23 s) (Figure 2). In contrast, the short-duration group showed no significant change between preflight (2.02 s, 1.53–2.17 s) and postflight (2.62 s, 1.89–3.79 s). While both groups performed similarly before flight, postflight times were significantly shorter in the short-duration group compared to the long-duration group (Table 1).

**TABLE 1:** Results of the mixed linear model. The model included subject group (Short, Long) and timepoint (Pre, Post) as fixed factors, and subject as a random factor. *p < 0.05.

	Fixed Effects					Pair hoc pairwise comparisons	
Sit-to-stand	Time to stand	Estimates	95% CI	*t-value*	*p-value*	Comparisons	*p-value*
	(intercept) Short vs. long Pre vs. post Group x Time	1.95 −0.08 2.69 −1.51	1.49 to 2.42 −1.07 to 0.89 2.03 to 3.34 −2.91 to −0.13	8.407 −0.177 8.165 −2.171	<0.001* 0.859 <0.001* 0.032*	Pre: long vs. short Post: long vs. short Long: pre vs. post Short: pre vs. post	0.859 0.002* <0.001* 0.061

Tandem walk – eyes open	% of correct steps	Estimates	95% CI	*t-value*	*p-value*	Comparisons	*p-value*
	(intercept) Short vs. long Pre vs. Post Group x Time	98.65 −1.82 −61.51 36.35	92.16 to 105.13 −15.73 to 12.09 −70.51 to −52.51 17.16 to 55.54	30.240 −0.260 −13.585 3.764	<0.001* 0.795 <0.001* <0.001*	Pre: long vs. short Post: long vs. short Long: pre vs. post Short: pre vs. post	0.795 <0.001* <0.001* 0.004*

Tandem walk – eyes closed	Time to complete	Estimates	95% CI	*t-value*	*p-value*	Comparisons	*p-value*
	(intercept) Short vs. long Pre vs. post Group x Time	76.79 5.04 −67.74 24.42	71.97 to 81.63 −5.09 to 15.18 −74.24 to −61.23 10.71 to 38.13	31.614 0.990 −20.711 3.541	<0.001* 0.325 <0.001* <0.001*	Pre: long vs. short Post: long vs. short Long: pre vs. post Short: pre vs. post	0.325 <0.001* <0.001* <0.001*

Walk-and-turn	Time to complete	Estimates	95% CI	*t-value*	*p-value*	Comparisons	*p-value*
	(intercept) Short vs. Long Pre vs. Post Group x time	9.10 0.54 18.19 −13.18	6.35 to 11.86 −4.81 to 5.89 14.44 to 21.93 −20.40 to −5.95	6.611 0.202 9.696 −3.643	<0.001* 0.841 <0.001* 0.001*	Pre: long vs. short Post: long vs. short Long: pre vs. post Short: pre vs. post	0.841 <0.001* <0.001* 0.109

Walk-and-turn	Turn rate	Estimates	95% CI	*t-value*	*p-value*	Comparisons	*p-value*
	(intercept) Short vs. long Pre vs. post Group x time	125.75 −5.18 −72.32 37.27	115.89 to 135.61 −24.34 to 13.99 −83.62 to −61.01 15.52 to 59.01	25.486 −0.540 −12.786 3.425	<0.001* 0.591 <0.001* 0.001*	Pre: long vs. short Post: long vs. short Long: pre vs. post Short: pre vs. post	0.591 0.001* <0.001* <0.001*

Motion sickness	Score	Estimates	95% CI	*t-value*	*p-value*		
	(intercept) Short vs. long	8.98 −4.36	6.68 to 11.28 −9.18 to 1.47	7.874 −1.280	<0.001* 0.076		

**FIGURE 3:**
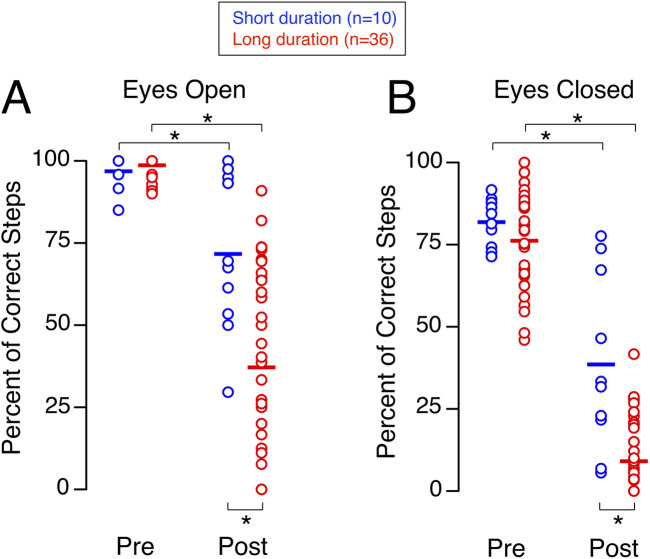
Tandem walk. Percentage of correct steps during the tandem-walk test with eyes open **(A)** and eyes closed **(B)** before (Pre) and shortly after (Post) spaceflight for 10 astronauts who participated in short-duration missions and 36 astronauts who participated in long-duration missions. Each symbol represents an individual subject, and the horizontal line indicates the group mean. *p < 0.05.

While performing the tandem walk test with their eyes open shortly after landing, both the short-duration group and the long-duration group made significantly less correct steps than they did before flight (Figure 3A). No significant difference in the percent of correct steps was detected between the short-duration group (100%, 94.78%–100%) and the long-duration group (100%, 100%–100%) before flight. In contrast, after flight, the percent of correct steps was significantly higher in the short-duration group (69.51%, 52.56%–95.63%) compared to the long-duration group (37.64%, 7.68%–64.16%) (Table 1).

The percent of correct steps while performing the tandem walk test with their eyes closed shortly after landing was significantly less than before flight for both the short-duration group and the long-duration group (Figure 3B). No significant difference in the percent of correct steps was detected between the short-duration group (82.89%, 73.86%–88.09%) and the long-duration group (79.31%, 64.81%–88.89%) before flight. In contrast, after flight, the percent of correct steps was significantly higher in the short-duration group (32.53%, 17.95%–68.41%) compared to the long-duration group (4.17%, 0.00%–19.21%) (Table 1).

**FIGURE 4:**
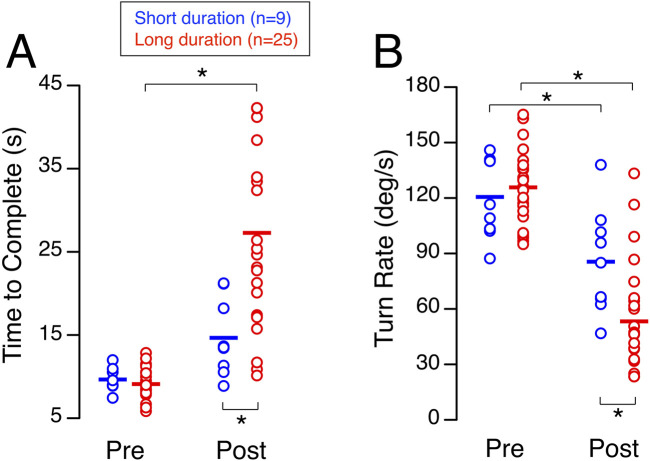
Walk-and-turn. Time to complete the obstacle course **(A)** and turn rate around the cone **(B)** during the walk-and-turn task before (Pre) and shortly after (Post) spaceflight for 9 astronauts who participated in short-duration missions and 25 astronauts who participated in long-duration missions. Each symbol represents an individual subject, and the horizontal line indicates the group mean. *p < 0.05.

Participants in the long-duration group required significantly more time to complete the walk-and-turn test shortly after landing (25.02 s, 17.29–40.51 s) than they did before flight (8.95 s, 8.15–10.21 s) (Figure 4A). In contrast, the short-duration group showed no significant change between preflight (8.91 s, 9.55–10.74 s) and postflight (13.51 s, 10.93–19.69 s). While both groups performed similarly before flight, after the flight the short-duration group completed the test significantly faster than the long-duration group (Table 1).

The average turn rate around the cone shortly after landing was significantly slower than before flight for both the short-duration group and the long-duration group (Figure 4B). No significant difference was detected for this measure between the short-duration group (116.52 deg/s, 102.78–140.44 deg/s) and the long-duration group (125.97 deg/s, 111.43–137.75 deg/s) before flight. In contrast, after flight, the short-duration group demonstrated a significantly faster turn rate compared to the long-duration group (84.99 deg/s, 64.09–104.79 deg/s vs. 46.29 deg/s, 26.72–65.53 deg/s) (Table 1).

**FIGURE 5:**
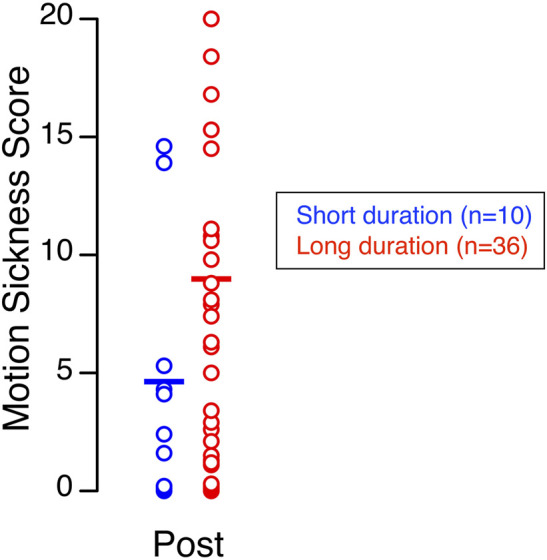
Terrestrial readaptation motion sickness. Motion sickness scores recorded shortly after landing for 10 astronauts who returned from short-duration missions and 36 astronauts who returned from long-duration missions. Each symbol represents an individual subject, and the horizontal line indicates the group mean.

Although the average subjective ratings of terrestrial readaptation motion sickness shortly after landing did not differ significantly between the long-duration group (7.98, 1.98–15.56) and the short-duration group (3.27, 0.00–7.48), the long-duration group exhibited a wider range of scores and a higher incidence of ratings above the midpoint (Figure 5; Table 1).

A linear mixed model analysis using timepoint (Pre, Post) and sex (Male, Female) as fixed factors revealed no significant main effects of sex on functional task performance or motion sickness scores.

## Discussion

Our study indicates that astronauts’ balance and locomotion is significantly impaired shortly after they return from short-duration spaceflight, as shown by the significant differences after flight relative to before flight for all the measures obtained during 3 functional tests, and by the presence of re-adaptation motion sickness symptoms shortly after landing. The decline in performance of functional tasks is characterized by increased time to achieve upright stance and complete an obstacle course, a reduced percentage of correct steps during tandem gait, and a decreased turn rate during locomotion after flight relative to pre-flight baseline measures. Short-duration space missions resulted in less decline in performance than long-duration missions. However, both long-duration and short-duration groups experienced similar levels of terrestrial readaptation motion sickness.

Differences in training protocols and exercise countermeasures across participant groups, along with the prior long-duration spaceflight experience of two short-duration crewmembers, may have impacted the observed results of this study.

The balance and locomotion tests used in our study challenge vestibular function, which is essential for performing critical mission tasks after transitions between gravity environments (Clément et al., 2022). Notably, individuals with bilateral vestibulopathy also exhibit impaired performance during the tests we used in this study (Clément et al., 2023). Previous studies employing posture platforms to assess astronauts after either short-duration Space Shuttle flights or long-duration ISS missions have repeatedly determined that astronauts have decreased postural stability (Clément et al., 1984; Paloski et al., 1993; Wood et al., 2015), which is consistent with our findings. Changes in postural strategies, particularly at the hip and ankle joints, have been reported after both short-duration (Speers et al., 1998) and long-duration (Shishkin et al., 2023) missions, with data collected the day after landing. The novelty of our study is the demonstration of a significant decline in walking and obstacle-avoidance performance within 3–4 h of landing, before substantial readaptation to Earth’s gravity could occur.

Previous studies have also shown that a majority of astronauts returning from long-duration ISS missions experience nausea, compared to approximately 15% following shorter Space Shuttle flights (Bacal et al., 2003; Reschke et al., 2017; Ramsburg et al., 2025). Jennings (1998) reported a greater incidence of motion sickness symptoms after longer duration Shuttle missions, and the higher range of symptom scores in the long-duration group is consistent with this trend.

Our results of the walk-and-turn test are also consistent with earlier research demonstrating adaptive changes in the coordination of eye, head, and trunk movements during locomotion shortly after return from short-duration Space Shuttle missions (Bloomberg et al., 1997; Newman et al., 1997). Turning around a target requires more cognitive effort after flight than before flight, which can impair motor performance. This suggests that processes such as computing self-rotation using somatosensory and vestibular cues and updating spatial orientation are disrupted by exposure to microgravity, regardless of its duration, and these processes must re-adapt after return to Earth (Glasauer et al., 1995).

When subjects tilt in a roll axis relative to gravity after returning from spaceflight, they tend to overestimate their tilt by approximately 30% (Clément et al., 2001; Clément and Wood, 2007; Clément and Wood, 2013a; Clément and Wood, 2013b; Clément and Wood, 2014). This heightened vestibular sensitivity contrasts with a post-flight reduction in ocular counter-rolling (Hallgren et al., 2016; Reschke et al., 2018; Schoenmaekers et al., 2022; Glukhikh et al., 2022). Interestingly, electrophysiological and anatomical studies in animals (Boyle et al., 2001; Ross, 2000) demonstrate that vestibular afferents exhibit increased sensitivity during re-adaptation to Earth’s gravity. This enhanced sensitivity of the otoliths is likely a major contributor to the postural instability, impaired locomotor performance, reduced turning speed, and re-entry motion sickness observed shortly after spaceflight (Clément et al., 2022).

The declines in posture and locomotion after short-duration ISS missions are comparable to those seen after Space Shuttle missions of similar length (Jennings, 1998), suggesting that these effects are not dependent of the spacecraft type or the launch and re-entry characteristics. Although individual responses vary, the overall performance impairments are generally less severe after short-duration missions compared to long-duration missions. Longer missions involve more substantial physiological changes—such as greater bone and muscle loss—that can compound sensorimotor disruptions and further impact performance. As a result, shorter missions may require fewer or less-intensive countermeasures than long-duration flights, allowing for faster recovery, simplified rehabilitation, and more efficient use of resources during re-adaptation to Earth’s gravity.

Maintaining stable posture, walking without vision, and navigating obstacles are tasks that private astronauts may encounter after returning to Earth, as well as Artemis astronauts during lunar landings following weightlessness. Successfully performing these tasks depends on the integration of visual, vestibular, and proprioceptive inputs, coordinated lower limb control, and cognitive functions such as spatial awareness and locomotor memory (Speers et al., 1998; Shishkin et al., 2023). Consequently, mitigation strategies will likely need to target not only vestibular impairments but also broader issues involving multi-sensory integration, movement coordination, and autonomic functions. Further research is needed to clarify the extent and focus of these countermeasures.

Limited physical activity during short-duration space missions may contribute to the declines in standing and walking performance observed in the short-duration group after flight. In contrast, astronauts on long-duration missions typically follow structured exercise protocols to help preserve muscle strength and function (English et al., 2020). However, current exercise regimens may not fully support optimal performance on functional tasks immediately after return to Earth (Clément et al., 2022). Incorporating targeted balance training could help improve post-landing stability during the critical first hours of recovery (Macaulay et al., 2021). Additional approaches worth exploring for both short-and long-duration missions include early post-flight rehabilitation, sensory augmentation, and combining non-pharmacological strategies with innovative delivery methods for anti-motion sickness medications.

## Data Availability

The datasets presented in this study can be found in online repositories. The names of the repository/repositories and accession number(s) can be found below: https://nlsp.nasa.gov/explore/page/home.
